# 2399: Protective immunity to live vaccines among children with solid tumors

**DOI:** 10.1017/cts.2017.230

**Published:** 2018-05-10

**Authors:** Franchesca Garcia Robles, Nilka DeJesus, Nilka Barrios

**Affiliations:** University of Puerto Rico-Medical Sciences Campus, San Juan, Puerto Rico

## Abstract

OBJECTIVES/SPECIFIC AIMS: Determine whether children with solid tumors maintain intact protective immunity to live vaccines during cancer therapy and after completing cancer therapy (postTx). METHODS/STUDY POPULATION: We will perform a prospective cohort study of children with solid tumors (Hodgkin lymphoma, brain, Wilms, and germ cell tumors) followed at the Puerto Rico’s University Pediatric Hospital. Protective immunity will be measured with antibody titers against live vaccines (Measles, Mumps, Rubella, and Varicella) at diagnosis, during cancer therapy, upon completion and 3 months postTx. RESULTS/ANTICIPATED RESULTS: We hypothesize that those patients with protective immunity to live vaccines prior to cancer therapy will lose it at the end of therapy. DISCUSSION/SIGNIFICANCE OF IMPACT: Loss of protective immunity to live vaccines has been reported in patients with hematologic malignancies after cancer therapy. This lack of protective immunity, which puts patients at higher risk of acquiring vaccine preventable diseases, has been limited studied in patients with solid tumors. The Center for Diseases Control has been established that it is safe to immunize cancer survivors with live vaccines 3 months post Tx. However, no clear guidelines for revaccination have been provided for this population. Understanding the protective immunity variation against live vaccines in children with solid tumors will allow us to identify the need for revaccination with live vaccines in this vulnerable population.

